# Molecular genetic portrait of virulence and ciprofloxacin resistance genes in clinical *Pseudomonas aeruginosa* Isolates from Khartoum, Sudan

**DOI:** 10.1371/journal.pone.0335269

**Published:** 2025-10-31

**Authors:** Azza Salah Imam Abdallah, Osama Mohamed, Maye M. Merghani, Musa Abdalla Ali

**Affiliations:** 1 Department of Microbiology, Faculty of Medical Laboratory Science, University of Khartoum, Khartoum, Sudan; 2 Exon Laboratory of Molecular Biology, Khartoum State, Sudan; 3 Department of Molecular Biology, National University Biomedical Research Institute, National University-Sudan, Khartoum, Sudan; 4 Nahda College, Khartoum, Sudan; Kampala International University - Western Campus, UGANDA

## Abstract

**Background:**

*Pseudomonas aeruginosa* remains a major cause of hospital- and community-acquired infections, with increasing ciprofloxacin resistance driven by mutations in quinolone resistance–determining regions (QRDRs) and plasmid-mediated mechanisms. This study aimed to determine the prevalence of key virulence genes *(oprI, toxA, lasB, nan1)* and ciprofloxacin resistance determinants *(gyrA, parC, qnrA, qnrB, qnrS*) in clinical isolates from Khartoum State, Sudan, and to explore associations with demographic and clinical variables.

**Methods:**

This cross-sectional study, which was conducted from January to April 2023, included eighty-six clinical isolates of *P.aeruginosa* that were collected from various hospitals in Khartoum State. The isolates were reidentified via standard microbiological techniques, and DNA was extracted via the boiling method. Multiplex polymerase chain reaction was utilized to detect the presence of virulence and ciprofloxacin resistance genes. Data analysis was performed via IBM SPSS software (version 20).

**Results:**

All the isolates carried one or more virulence genes, with oprI being the most prevalent (88.4%), followed by *lasB* (80.2%), *toxA* (57%), and nan1 (6.98%). Among the isolates, 30 (34.9%) were resistant to ciprofloxacin, whereas 56 (65.1%) were susceptible. All resistant isolates carried at least one of the resistance genes studied. The *parC* gene was the most prevalent (40.7%), followed by *gyrA* (20.9%) and *qnrS* (19.8%). *qnrA* and *qnrB* each had a prevalence of 17.4%. This investigation revealed the coexistence of the *gyrA* and *parC* genes in seven isolates (23.3%), and we also reported that the *qnrA*, *qnrB,* and *qnrS* genes coexisted in 11 (36.7%) of the ciprofloxacin resistant *P. aeruginosa* isolates. A significant association was detected between ciprofloxacin resistance and the presence of the *gyrA*, *qnrS*, *qnrA*, and *qnrB* genes (p < 0.001) but not the *parC* gene (p = 0.6). There was no significant association between ciprofloxacin resistance genes and virulence genes (p > 0.05).

**Conclusions:**

The prudent use of ciprofloxacin is vital in managing *P.aeruginosa* infections amid rising resistance. Detection of *gyrA* and *parC* in susceptible isolates signals potential for future resistance through future mutations, highlighting the need for ongoing monitoring. The coexistence of resistance and virulence genes highlights the pathogen’s combined threat. These findings reinforce the public health importance of continuous molecular surveillance and genetic profiling, not only to guide effective treatment but also to inform targeted infection control strategies and antimicrobial stewardship programs.

## Introduction

*Pseudomonas aeruginosa* is a Gram-negative bacterium commonly found in places like soil and water, as well as on hospital surfaces and medical devices [1]. Although it rarely causes illness in healthy people, it can pose a serious risk to those with weakened immune systems. *P. aeruginosa* is a common source of serious healthcare-associated infections such as ventilator-acquired pneumonia, urinary tract infections, bloodstream infections, and surgical wound infections.

*P. aeruginosa* can be particularly dangerous for people with cystic fibrosis because it often causes long-lasting lung infections that are hard to treat, making it harder for patients to stay healthy and impacting their overall quality of life and long-term outlook. Its ability to quickly develop resistance to many antibiotics makes managing these infections even more challenging for doctors. It occurs through a variety of mechanisms, including biofilm formation, active efflux pumps, and the production of drug-inactivating enzymes [1]. What makes things even more complicated is *P. aeruginosa’s* genetic flexibility, which means it can easily pick up new resistance genes including those that cause resistance to fluoroquinolones such as ciprofloxacin, like mutations in *gyrA* and *parC* [2]. This makes treating these infections even more challenging. These features have led to its classification as a critical-priority pathogen by global health authorities, emphasizing the urgent need for improved infection control measures and novel therapy alternatives [1,3]

In *P.aeruginosa*, ciprofloxacin resistance arises through three main mechanisms: mutations in quinolone resistance-determining regions (QRDRs), such as DNA gyrase (*gyrA*) and topoisomerase IV (*parC*); increased membrane permeability due to the upregulation of efflux pumps; and the presence of plasmid-mediated quinolone resistance genes (PMQRs) [2]. PMQR includes various mechanisms, such as quinolone resistance proteins (encoded by qnr genes such as *qnrA*, *qnrB*, *qnrC, qnrD*, and *qnrS*), which protect DNA gyrase and topoisomerase IV from the effects of fluoroquinolones (FQs) [4].

*The* pathogenicity of *P.aeruginosa* is due to various extracellular and cell-associated virulence factors. Examples of genes that encode and contribute to these virulence factors include *toxA*, *oprI, lasB,* and *nan1*. Exotoxin A, encoded by the *toxA* gene, inhibits protein production in host cells and generates redox-active phenazines that are toxic to human cells. [5]. The *lasB* gene encodes elastase B, a zinc-dependent metalloprotease that plays a key role in the virulence of *P. aeruginosa*. The presence of *lasB* is commonly associated with increased tissue damage and immune system evasion, contributing significantly to the pathogen’s ability to cause severe infections. Its activity supports the persistence and spreads of *P. aeruginosa* in both acute and chronic infections, making it a critical factor in the organism’s pathogenic profile [6]. Neuraminidase, produced by the nan1 gene, aids in bacterial adhesion to epithelial cells. Additionally, the *oprI* genes in the outer membrane facilitate the efficient detection of *Pseudomonas aeruginosa*. [7].

Key virulence genes (*oprL, oprI, lasB, toxA, nan1*) and resistance genes (*gyrA, parC, qnrA, qnrB, qnrS*) were chosen because of their well-known importance in clinical settings. The selected virulence genes play roles in helping the bacteria evade the immune system, cause tissue damage, and establish infections in the body. The resistance genes, on the other hand, are linked to either chromosomal mutations or plasmid-mediated mechanisms that give the bacteria resistance to ciprofloxacin. Their inclusion was also driven by the limited molecular data available on *P. aeruginosa* isolates from Sudan. By studying these isolates, this work aims to help fill that gap and shed light on the local patterns of virulence and resistance genes.

We hypothesized that clinical isolates of *Pseudomonas aeruginosa* from Khartoum would carry a variety of virulence and ciprofloxacin resistance genes. We also thought that ciprofloxacin resistance would be tied to certain resistance genes, and that the presence of virulence genes might also have an impact possibly making these bacteria more capable of causing disease. Additionally, we aimed to identify the most prevalent virulence and ciprofloxacin resistance genes in all *P.aeruginosa* isolates. Given what’s been shown in previous studies and what we found, we expected that virulence genes wouldn’t have a strong link to resistance.

The purpose of this study was to detect and determine the prevalence of virulence genes (***t****oxA*, *lasB*, *nan1*, and *oprI*), as well as resistance genes (*gyrA, parC, qnrA*, *qnrB*, and *qnrS*), and to determine whether there is an association between ciprofloxacin resistance and the detection of resistance and virulence genes in *P. aeruginosa* strains.

## Materials and methods

### Study design and sample collection

This study was a descriptive cross-sectional study of *P. aeruginosa* isolates isolated from various clinical samples, including urine, wound swabs, sputum, ear swabs, blood cultures, tracheal aspirates, eye swabs, pus, cough swabs, and tissue and body fluid samples collected between

January 2023 and April 2023 from hospitals in Khartoum State, Sudan. Only non-duplicate *P. aeruginosa* isolates from both hospitalized patients and outpatients were included. Isolates were confirmed as *P. aeruginosa* using standard biochemical tests. The sampling period may have been influenced by environmental factors that affect the prevalence and resistance patterns of bacterial pathogens. Samples were included regardless of the infection site to ensure a broad representation of circulating strains. Environmental samples, surveillance specimens, repeat isolates from the same patient, and isolates lacking essential metadata (e.g., source or antibiotic susceptibility profile) were excluded.

### Phenotypic characterization

A standard scheme for identifying all *Pseudomonas aeruginosa* strains was used [8]. Clinical isolates of *P. aeruginosa* were subcultured on nutrient agar and MacConkey agar, then incubated at 37°C for 24 hours to ensure purity and optimal growth.

Gram-negative bacteria were isolated, and non-lactose fermenting organisms were distinguished via MacConkey agar media. Colonial morphology and pigment formation were distinguished on nutrient agar. Gram-negative rods with positive oxidase tests were identified via conventional biochemical tests (KIA, urease, citrate, motility and indole). All these reagents were imported from Oxoid (Bactident Co.). [8]

### Antimicrobial susceptibility testing

Ciprofloxacin (5 μg) susceptibility testing was performed on all the verified *P. aeruginosa* isolates. It was examined on Mueller‒Hinton agar via the Kirby‒Bauer disk diffusion method. We placed ciprofloxacin disks on each inoculated Mueller‒Hinton agar plate and incubated them at 37°C for 24 hours. The plates were examined for zones of inhibition around the disk. These strains were evaluated and compared to an interpretation table to determine sensitive and resistant strains. [8]

### Multiplex PCR for the detection of virulence genes (*toxA, oprI, nan1* and *lasB*) and antibiotic resistance genes (*gyrA, parC, qnrA, qnrB & qnrS*)

#### DNA extraction.

Bacterial DNA was extracted from pure cultures of *P. aeruginosa* isolates via the boiling method. Briefly, 2–3 colonies of a pure culture of *P. aeruginosa* were suspended in 50 *µ*L of nuclease-free water. The suspension was heated to 95°C for 10 minutes, incubated at −20°C for 10 minutes, and then centrifuged at 10,000 rpm for 5 minutes. 5 *µ*L of the supernatant was used as a template in the PCR assay [9].

#### Multiplex PCR for detection of virulence and resistance genes.

Multiplex PCR was conducted to confirm the presence of *P. aeruginosa* resistance and virulence genes via specific primers in three tubes: first, *toxA, oprI, nan1* and *lasB*; second, g*yrA and parC;* and finally, q*nrA, qnrB* and *qnrS*. The reaction mixture was 20 μl in total, containing 4 μl of a mixture with Taq polymerase, reaction buffer, MgCl₂, and dNTPs (Solis Biodyne, Estonia), 1 μl each of forward and reverse primers (10 pmol/μl) for the target genes, 5 μl of DNA template, and water to reach a final volume of 20 μl. PCR was performed with the following thermal cycling conditions: initial denaturation at 95°C for 5 minutes; 35 cycles of denaturation at 95°C for 1 minute, annealing at 60°C for 1 minute, and extension at 72°C for 1 minute; followed by a final extension at 72°C for 5 minutes.. The PCR products were analysed using agarose gel electrophoresis, stained with ethidium bromide visualized under UV light, and compared to a 100 bp DNA ladder using the Bio-Rad Gel Doc System. The National Public Health Laboratory (NPHL), Department of Microbiology, Sudan, prepared and provided a mixed positive control consisting of known *P. aeruginosa* strains harboring target resistance and virulence genes. To ensure assay specificity and rule out contamination, each PCR run included both a positive control and a no-template negative control. These controls were essential for validating the accuracy and reliability of the amplification results. The primers used for each gene are listed in Table 1 and Table 2.

**Table 1:** Sequences of Primers used for the detection of virulence genes in *P*.*aeruginosa* isolates: This table lists the specific oligonucleotide primer sequences used to amplify target virulence genes in Pseudomonas aeruginosa clinical isolates. Forward (F) and reverse (R) sequences are given in the 5′–3′ direction. Product sizes are listed in base pairs (bp). All primer sets were adopted from a previous study [10].

Amplified target gene	Specific Primer sequence (5′–3′)	Product size (bp)	Reference
*oprI*	F: ATGAACAACGTT CTG AAA TTC TCT GCT R: CTT GCG GCT GGC TTT TTC CAG	250	[10]
*toxA*	F: GGT AAC CAG CTC AGC CAC AT R: TGA TGT CCA GGT CAT GCT TC	352	[10]
*lasB*	F: GGA ATG AAC GAA GCG TTC TC R: GGT CCA GTA GTA GCG GTT GG	300	[10]
*nan1*	F: ATG AAT ACT TAT TTT GATAT R: CTA AAT CCA TGC TCTGACCC	1316	[10]

• F: Forward primer.

• R: reverse primer.

• bp: base pair.

**Table 2:** Sequences of Primers Used for the Detection of ciprofloxacin resistance genes in P. aeruginosa Isolates: This table lists the specific primer sequences (5′–3′) used to detect resistance genes in *P. aeruginosa.*

Amplified gene	Primer sequence (5′–3′)	Product size (bp)	reference
gyrA	F: CGATTCCGGCTTCCGCCCGG	194	[11]
	R: CCGGTGGGTCA TTGCCTGGCG		
parC	F: AAACCTGTTCAGCGCCGCATT	395	[12]
	R: GTGGTGCCGTTAAGCAAA		
qnrA	F: ATTTCTCACGCCAGGATTTG	516	[13]
	R: GATCGGCAAAGGTTAGGTCA		
qnrB	F: GATCGTGAAAGCCAGAAAGG	476	[13]
	R: ATGAGCAACGATGCCTGGTA		
qnrS	F: GCAAGTTCATTGAACAGGGT	428	[13]
	R: TCTAAACCGTCGAGTTCGGCG		

• F: Forward primer

• R: reverse primer.

• bp: base pair.

### Statistical analysis

All statistical analyses were performed using IBM SPSS Statistics version 20 (IBM Corp., Armonk, NY), selected for its reliability and suitability for categorical data analysis and logistic regression. The chi-square test was applied to assess associations between categorical variables, such as the presence of resistance or virulence genes and ciprofloxacin susceptibility. To address the issue of multiple testing, we applied the Benjamini–Hochberg false discovery rate (FDR) correction, setting the significance threshold (Q) at 0.05. We reported FDR-adjusted p-values and considered any results with adjusted p-values less than 0.05 to be statistically significant.

We described the distribution of resistance and virulence genes, as well as CIP susceptibility, using basic descriptive statistics. For categorical variables, we reported frequencies and percentages. To better understand the findings, we calculated odds ratios (ORs) with 95% confidence intervals (CIs) as our measure of effect size. However, for genes that were extremely rare or had the same result across all samples (like the qnr genes), we reported relative risks (RRs) instead. To explore things in more depth, we built a multivariable binary logistic regression model to examine the independent effects of specific genes on CIP resistance. We included all genes that showed enough variability. Results are shown as FDR-adjusted p-values, together with the corresponding ORs and 95% CIs. Genes that were only present in CIP-resistant isolates and absent in all sensitive ones couldn’t be included, since it wasn’t possible to statistically model their effects.

### Ethics statement

This study was approved by the Ethical Review Committee at the National University Biomedical Research Institute in Khartoum, Sudan (Approval Number: NU-RECG276) in January 2023. The research was originally titled “Genetic Portrait of Virulence and Resistance in *Pseudomonas aeruginosa* Isolates from Khartoum, Sudan.” The title was later updated to “ Molecular Genetic Portrait of Virulence and Ciprofloxacin Resistance Genes in Clinical Pseudomonas aeruginosa Isolates from Khartoum, Sudan “ to enhance clarity and specificity. We obtained written informed consent from all adult participants and from parents or legal guardians for minors before the study began. The research followed the principles of the Declaration of Helsinki. Participation was voluntary, with samples collected as part of routine clinical care and minimal risk to participants. We took care to maintain strict confidentiality for everyone involved.

## Result

A total of 86 non duplicate *P. aeruginosa* isolates were collected from different clinical samples in several hospitals throughout Khartoum State, Sudan. Most of these isolates came from urine samples (31 cases, 36.0%), followed by wound swabs (17 cases, 19.7%), sputum samples (12 cases, 14.0%), and ear swabs (9 cases, 10.5%). The remaining isolates were obtained from blood, tracheal aspirates, eye swabs, pus, and tissue samples (see Table 3).Regarding hospital distribution, Soba University Hospital contributed the highest number of isolates (n = 59, 68.6%), followed by Fedail Hospital (n = 9, 10.5%), Royal Hospital (n = 6, 7.0%), and other participating hospitals, including Military, Bahri, and Alribat (Table 4). The samples were collected from a variety of hospital units, with the largest share coming from the Intensive Care Unit (ICU) and its different sections. In total, 33 isolates (38.4%) were from the general ICU, 15 (17.4%) from the male ICU, and 5 (5.8%) from the ICU specializing in respiratory therapy. The outpatient departments contributed 13 isolates (15.1%), while 7 (8.1%) came from the Emergency Room. Additional isolates were gathered from the Neonatal Intensive Care Unit (NICU), Pediatric Unit, and surgical wards (see Table 5).

**Table 3:** Distribution of *Pseudomonas aeruginosa* isolates by clinical sample type (n = 86): This table summarizes the frequency and percentage distribution of clinical specimens from which *Pseudomonas aeruginosa* isolates were obtained.

Sample type	Frequency	Percent
Blood	5	5.8
Ear Swab	9	10.5
Eye swab	2	2.3
Wound Swab	17	19.7
Sputum	12	14.0
Tracheal Aspirate	4	4.7
Urine	31	36.0
Cough Swab	1	1.2
Fluid	1	1.2
Pus	2	2.2
RF	1	1.2
TISSUE	1	1.2
Total	86	100.0

*RF: Renal fluid.

**Table 4:** Distribution of *Pseudomonas aeruginosa* isolates by hospital (n = 86): This table presents the number and percentage of *P. aeruginosa* isolates obtained from different hospitals.

Hospital	Frequency	Percent
Soba	59	68.6
Fedail	9	10.5
Royal	6	7.0
Military	5	5.8
Bahri	4	4.6
Alribat	3	3.5
Total	86	100.0

Percentages were calculated based on the total number of *P. aeruginosa* isolates (n = 86).

**Table 5:** Distribution of *Pseudomonas aeruginosa* isolates by hospital unit (n = 86). This table shows the distribution of *P. aeruginosa* clinical isolates according to hospital.

Units	Frequency	Percent
ICU	33	38.4
ICU-M	15	17.4
OUT	13	15.1
ER	7	8.1
ICU-RT	5	5.8
ICU-ERP	3	3.5
ICU-F	2	2.3
NICU	2	2.3
Pediatric	2	2.3
OR	1	1.2
PICU	1	1.2
PICU-RT	1	1.2
ICU-CNT	1	1.2
Total	86	100.0

Notes:

ICU: Intensive Care Unit; ICU-M: ICU–Medical; ICU-RT: ICU–Respiratory Therapy; ICU-ERP: ICU–Emergency Respiratory Patients; ICU-F: ICU–Female; ICU-CNT: ICU–Contaminated;

NICU: Neonatal ICU; PICU: Pediatric ICU; PICU-RT: PICU–Respiratory Therapy;

ER: Emergency Room; OR: Operating Room; OUT: Outpatient;

Percentages calculated based on the total number of isolates (n = 86).

### Ciprofloxacin susceptibility patterns

A total of 86 *P.aeruginosa* samples were tested to see how they responded to ciprofloxacin. Out of these, 30 samples (34.9%) showed resistance to the antibiotic, while 56 (65.1%) were still susceptible. The samples were collected from both male (50) and female (36) patients.

### Association with patient gender

This study examined whether there was a connection between a patient’s gender and their resistance to ciprofloxacin. While resistance was a bit more common among male patients (60%) than female patients (40%), this difference wasn’t statistically significant (P = 0.798). More details about the distribution and odds ratio can be found in Table 6.

**Table 6:** Association between gender and ciprofloxacin (CIP) resistance among *Pseudomonas aeruginosa* isolates (n = 86).

Gender	CIP Sensitive, n (%)	CIP Resistant, n (%)	P. value	Odds Ratio (95% CI)
Male	32 (57.1%)	18 (60.0%)	0.798	0.889 (0.361–2.191)
Female²	24 (42.9%)	12 (40.0%)		

### Association with Hospital units

A significant association was found between hospital unit and ciprofloxacin (CIP) resistance among *Pseudomonas aeruginosa* isolates (p = 0.015) (Table 7). The highest proportion of ciprofloxacin-sensitive isolates was recovered from the ICU (35.7%) and the outpatient unit (23.2%), while ciprofloxacin-resistant strains were predominantly found in the ICU (43.3%) and ICU-M (30.0%). Notably, no ciprofloxacin-resistant isolates were observed in the emergency room (ER), outpatient (OUT), pediatric, or operating room (OR) units. Conversely, resistant strains were identified in ICU subunits such as ICU-ERP (6.7%), ICU-RT (10.0%), ICU-CNT (3.3%), and PICU-RT (3.3%), suggesting a concentration of resistance in intensive and critical care settings.

**Table 7:** Association between hospital units and ciprofloxacin (CIP) resistance in *P.aeruginosa* isolates.

Hospital Unit	CIP-Sensitive, n (%)	CIP-Resistant, n (%)	P-value¹
ER	7 (12.5%)	0 (0.0%)	0.015*
ICU	20 (35.7%)	13 (43.3%)	
ICU-CNT	0 (0.0%)	1 (3.3%)	
ICU-ERP	1 (1.8%)	2 (6.7%)	
ICU-F	2 (3.6%)	0 (0.0%)	
ICU-M	6 (10.7%)	9 (30.0%)	
ICU-RT	2 (3.6%)	3 (10.0%)	
NICU	1 (1.8%)	1 (3.3%)	
OR	1 (1.8%)	0 (0.0%)	
OUT	13 (23.2%)	0 (0.0%)	
Pediatric	2 (3.6%)	0 (0.0%)	
PICU	1 (1.8%)	0 (0.0%)	
PICU-RT	0 (0.0%)	1 (3.3%)	

Notes:

¹ P-value calculated using the chi-square test. *Statistically significant at p < 0.05.

ICU: Intensive Care Unit; ICU-M: ICU–Medical; ICU-RT: ICU–Respiratory Therapy; ICU-ERP: ICU–Emergency Respiratory Patients; ICU-F: ICU – Female Ward, ICU-CNT: ICU – Contaminated Ward;

NICU: Neonatal ICU; PICU: Pediatric ICU; PICU-RT: PICU–Respiratory Therapy;

ER: Emergency Room; OR: Operating Room; OUT: Outpatient.

### Multiplex PCR for virulence gene detection

**Fig 1:**
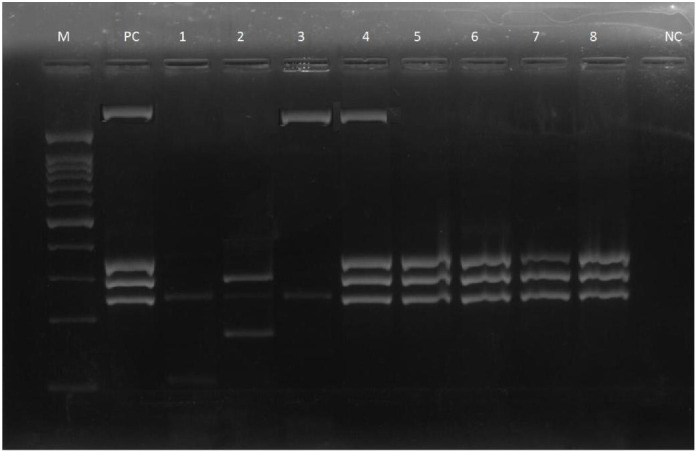
Original gel electrophoresis image showing PCR amplification of virulence genes (oprI, lasB, toxA, and nan1) in Pseudomonas aeruginosa. Lane M: DNA ladder (marker); Pc: positive control; Nc: negative control. This unprocessed image was used to confirm the presence of virulence genes by multiplex PCR. The raw gel image is available at Zenodo: https://doi.org/10.5281/zenodo.16413982.

**Fig 2:**
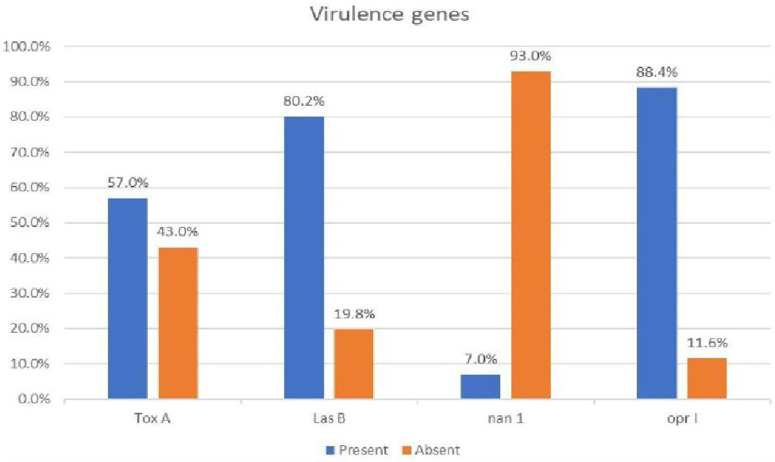
Prevalence of virulence genes among Pseudomonas aeruginosa isolates. The bar chart shows the proportion of isolates positive (blue) and negative (orange) for each tested virulence gene (toxA, lasB, nan1, oprI). Prevalence is expressed as a percentage of the total 86 isolates tested.

All the isolates possessed one or a combination of virulence genes, except for one isolate Fig 1. *OprI* was the most prevalent virulence gene; it was detected in 76 (88.4%) of the isolates; the *lasB* gene was detected in 69 (80.2%) isolates; the *toxA* gene was detected in 49 (57%) isolates; and the *nan* 1 gene was detected in 6 (6.98%) isolates. (**Fig 2**). Representative amplification results are shown in **Fig 1**.

### Multiplex PCR for resistance gene detection

**Fig 3:**
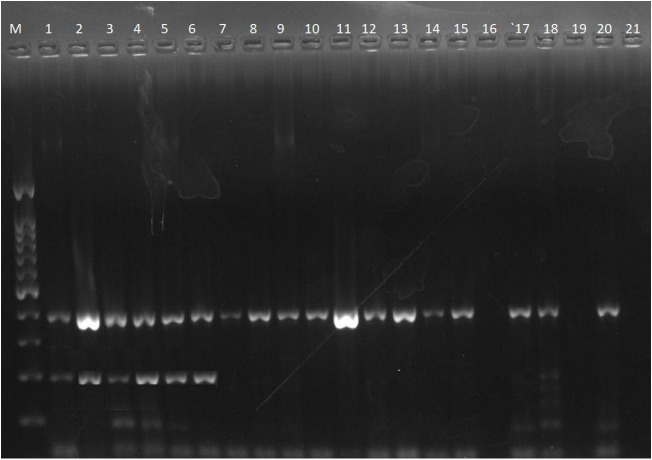
Gel electrophoresis of the PCR products of theamplification of gyrA and parC resistance genes (194 and 395 bp, respectively) in P.aeruginosa isolates. M: Molecular marker (100 bp). The Original Gel electrophoresis image of the PCR products of the amplification of the gyrA and parC ciprofloxacin resistance genes in p.aeruginosa. Zenodo. https://doi.org/10.5281/zenodo.16739094.

**Fig 4:**
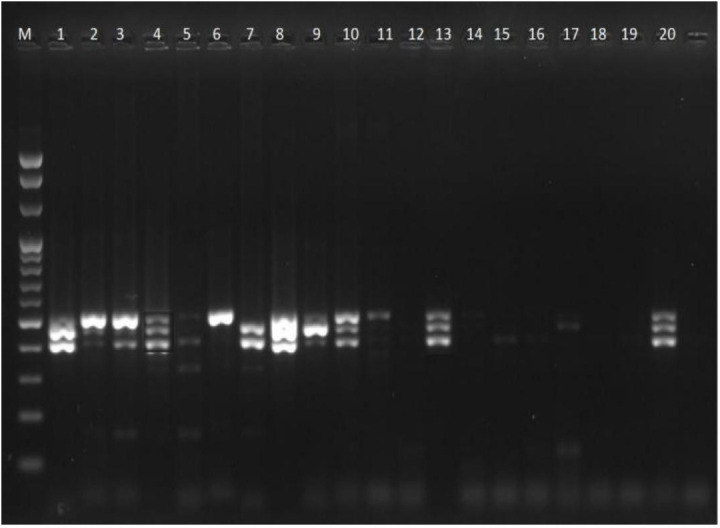
Gel electrophoresis of the PCR products of theamplification of qnrA, qnrB, and qnrS resistance genes (516 bp, 476 bp, and 428 bp, respectively) in P.aeruginosa isolates. M = Molecular marker (100 bp). The gel shows the PCR amplification results for the qnrA, qnrB, and qnrS ciprofloxacin resistance genes in Pseudomonas aeruginosa isolates. The original gel electrophoresis image is available at Zenodo: https://doi.org/10.5281/zenodo.16485795.

Multiplex PCR was performed to detect QRDR genes in *P. aeruginosa* isolates. The amplified products of the *gyrA* and *parC* genes (194 bp and 395 bp, respectively) are shown in Fig 3. Similarly, amplification of the *qnrA* (516 bp), *qnrB* (476 bp), and *qnrS* (428 bp) genes is demonstrated in Fig 4. All the isolates that were resistant to ciprofloxacin harboured either one or a mixture of resistance genes.

**Fig 5:**
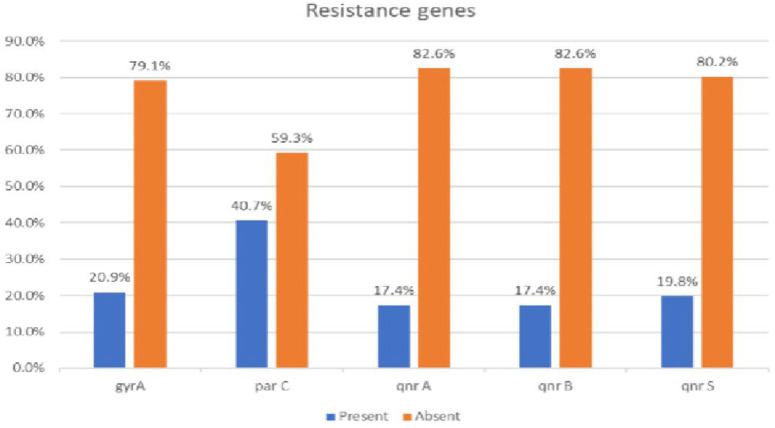
Prevalence of resistance genes among P. aeruginosa isolates. The bar chart shows the proportion of isolates that tested positive (blue) and negative (orange) for each of the ciprofloxacin resistance genes: gyrA, parC, qnrA, qnrB, and qnrS. Prevalence is expressed as a percentage of the total number of isolates tested (n = 86).

Among all the 86 *P. aeruginosa* isolates, *parC* was the most prevalent resistance gene, detected in 35 isolates (40.7%), followed by *gyrA* in 18 isolates (20.9%), *qnrS* in 17 isolates (19.8%), and both *qnrA* and *qnrB* in 15 isolates each (17.4%) as demonstrated in Fig 5.

This investigation revealed the coexistence of the *gyrA* and *parC* genes in seven (23.3%) of the *P.aeruginosa* isolates. We also found that the PMQR genes under study (*gnrA*, *qnrB*, and *qnrS*) coexisted in 11 (36.7%) of all *P. aeruginosa* isolates.

### Association between resistance genes and ciprofloxacin resistance

After performing Pearson’s chi-square test, followed by Benjamini–Hochberg (FDR) correction with a significance threshold set at Q = 0.05 for the possibility of false positives, we found that the genes *gyrA, qnrA, qnrB,* and *qnrS* were all strongly associated with CIP resistance(P < 0.001 for all). These associations remained significant even after further statistical corrections, indicating a strong connection between these genes and resistance to the ciprofloxacin. In contrast, we did not find any significant link between the *parC* gene and CIP resistance (P = 0.600). More detailed data and effect estimates can be found in Tables 8 and 9.

**Table 8:** Association between resistance genes and ciprofloxacin (CIP) resistance in *P.aeruginosa* isolates.

Gene	Status	CIP Sensitive (N = 56)	CIP Resistant (N = 30)	P-value	BH Adjusted P-value	sEffect Estimate (95% CI)
gyrA	Present Absent	2 (3.6%) 54 (96.4%)	16 (53%) 14 (47%)	<0.001	0.0056*	OR = 0.032 (0.007–0.158)
ParC	Present Absent	24 (43%) 32 (57%)	11 (37%) 19 (63%)	0.600	0.0444*	OR = 1.295 (0.520–3.224)
qnrA	Present Absent	0 (0%) 56 (100%)	15 (50%) 15 (50%)	<0.001	0.0111*	RR = 4.733 (3.020–7.419)
qnrB	Present Absent	0 (0%) 56 (100%)	15 (50%) 15 (50%)	<0.001	0.0167*	RR = 4.733 (3.020–7.419)
qnrS	Present Absent	0 (0%) 56 (100%)	17 (57%) 13 (43%)	<0.001	0.0222*	RR = 5.308 (3.253–8.661)

¹ P-values were calculated using the Chi-square test or Fisher’s exact test, as appropriate. Multiple testing correction was applied using the Benjamini-Hochberg (BH) procedure. Effect estimates are expressed as odds ratios (OR) or relative risks (RR) with 95% confidence intervals (CI). CIP, ciprofloxacin; OR odds ratio; RR, relative risk; CI, confidence interval. Statistically significant BH-adjusted P-values (P < 0.05) are indicated with an asterisk (*).

**Table 9:** Association Between virulence Genes and Ciprofloxacin Resistance.

Gene	Status	CIP Sensitive (N = 56)	CIP Resistant	P-value	BH Adjusted P-value	Effect Estimate (95% CI)
ToxA	Present Absent	30 (53.6%) 26(46.4%)	19(63.3%) 11(36.7%)	0.384	0.0333	OR = 0.668 (0.269–1.659)
LasB	Present Absent	44 (78.6%) 12(21.4%)	25(83.3%) 5(16.7%)	0.597	0.0389	OR = 0.733 (0.232–2.323)
nan1	Present Absent	2 (3.6%) 54 (96.4%)	4 (13.3%) 26(86.7%)	0.090	0.0278	OR = 0.241 (0.041–1.400)
OprI	Present Absent	49 (87.5%) 7 (12.5%)	27(90.0%) 3 (10.0%)	0.730	0.0500	OR = 0.778 (0.186–3.256)

• *P-values calculated using Pearson’s chi-square test.*

• ***BH ****= Benjamini–Hochberg adjusted p-value (False Discovery Rate correction at Q = 0.05).*

• ***OR*** *= Odds Ratio;* ***RR ****= Relative Risk (used where odds ratio is undefined due to 0 values).*

### Logistic regression analysis of gene associations with ciprofloxacin resistance

Logistic regression was used to assess the association between individual genes and ciprofloxacin (CIP) resistance. After applying Benjamini–Hochberg correction for multiple comparisons (FDR), only *gyrA* remained statistically significant (adjusted P < 0.001), showing a strong association with resistance (OR = 33.535; 95% CI: 5.974–188.237) as illustrated in Table 10. None of the tested virulence genes (*toxA*, *lasB*, *nan1*, and *oprI*) showed a statistically significant association with CIP resistance (adjusted P > 0.05). The genes *qnrA*, *qnrB*, and *qnrS* were excluded from the logistic regression analysis due to lack of variability; they were absent in all CIP-sensitive isolates (100%), preventing model estimation.

**Table 10:** Logistic regression analysis of gene associations with ciprofloxacin (CIP) resistance after Benjamini–Hochberg (FDR) correction: Regression results include unadjusted and adjusted p-values, odds ratios (OR), and 95% confidence intervals (CI) for each gene. Only *gyrA* remained significantly associated after correction.

Gene	P-value	BH Adjusted P-value	Odds Ratio (OR)	95% Confidence Interval (CI)
*toxA*	0.642	0.664	1.443	0.308–6.759
*lasB*	0.664	0.664	1.535	0.222–10.633
*nan1*	0.541	0.664	2.407	0.144–40.347
*oprI*	0.458	0.664	2.532	0.218–29.426
*gyrA*	<0.001	<0.001*	33.535	5.974–188.237
*parC*	0.443	0.664	0.629	0.192–2.057

Notes:

OR: Odds Ratio; CI: Confidence Interval; BH: Benjamini–Hochberg (False Discovery Rate adjustment (FDR) method).

*Statistically significant at adjusted p < 0.05.

### Distribution of resistance and virulence genes by sample type

The types of resistance and virulence genes we found in *P. aeruginosa* depended on where the samples came from (Table 11). In urine samples—the largest group in our study—the most common virulence genes were *oprI* (23.5%), *lasB* (20.0%), and *toxA* (13.0%), The *nan1* gene was present at a lower frequency (**3.5%**). while *parC* (11.3%), *gyrA* (10.4%), and *qnrA* (7.0%) were the main resistance genes.

**Table 11:** Distribution of Resistance and Virulence Genes by Sample Type in *Pseudomonas aeruginosa* Clinical Isolates: This table shows the frequency and percentage of each tested virulence gene (*toxA, lasB, nan1, oprI*) and resistance gene (g*yrA, parC, qnrA, qnrB, qnrS)* across clinical specimen types. Values are expressed as n (%).

Sample Type	*ToxA*	*LasB*	*nan1*	*oprI*	*gyrA*	*parC*	*qnrA*	*qnrB*	*qnrS*
Blood	3 (18.8%)	4 (25.0%)	1 (6.2%)	4 (25.0%)	1 (6.2%)	3 (18.8%)	0 (0.0%)	0 (0.0%)	0 (0.0%)
Ear Swab	5 (13.5%)	7 (18.9%)	0 (0.0%)	9 (24.3%)	1 (2.7%)	5 (13.5%)	3 (8.1%)	3 (8.1%)	4 (10.8%)
Eye Swab	1 (20.0%)	1 (20.0%)	0 (0.0%)	2 (40.0%)	0 (0.0%)	1 (20.0%)	0 (0.0%)	0 (0.0%)	0 (0.0%)
Wound Swab	11 (21.2%)	16 (30.8%)	0 (0.0%)	14 (26.9%)	2 (3.8%)	6 (11.5%)	1 (1.9%)	1 (1.9%)	1 (1.9%)
Sputum	9 (20.0%)	11 (24.4%)	0 (0.0%)	10 (22.2%)	2 (4.4%)	5 (11.1%)	3 (6.7%)	3 (6.7%)	2 (4.4%)
Tracheal Aspirate	1 (8.3%)	2 (16.7%)	1 (8.3%)	4 (33.3%)	0 (0.0%)	2 (16.7%)	0 (0.0%)	1 (8.3%)	1 (8.3%)
Urine	15 (13.0%)	23 (20.0%)	4 (3.5%)	27 (23.5%)	12 (10.4%)	13 (11.3%)	8 (7.0%)	6 (5.2%)	7 (6.1%)
Cough Swab	1 (33.3%)	1 (33.3%)	0 (0.0%)	1 (33.3%)	0 (0.0%)	0 (0.0%)	0 (0.0%)	0 (0.0%)	0 (0.0%)
Fluid	0 (0.0%)	1 (50.0%)	0 (0.0%)	1 (50.0%)	0 (0.0%)	0 (0.0%)	0 (0.0%)	0 (0.0%)	0 (0.0%)
Pus	1 (20.0%)	1 (20.0%)	0 (0.0%)	2 (40.0%)	0 (0.0%)	0 (0.0%)	0 (0.0%)	0 (0.0%)	1 (20.0%)
RF	1 (20.0%)	1 (20.0%)	0 (0.0%)	1 (20.0%)	0 (0.0%)	0 (0.0%)	0 (0.0%)	1 (20.0%)	1 (20.0%)
Tissue	1 (33.3%)	1 (33.3%)	0 (0.0%)	1 (33.3%)	0 (0.0%)	0 (0.0%)	0 (0.0%)	0 (0.0%)	0 (0.0%)

• RF = body fluid (e.g., pleural/ascitic fluid).

• **nan1* *= neuraminidase gene.

• **oprI* *= outer membrane lipoprotein I.

• qnr = plasmid-mediated quinolone resistance gene.

• Percentages calculated based on total number of isolates per sample type.

For wound swabs, *lasB* (30.8%), *oprI* (26.9%), and *toxA* (21.2%) were again the most frequently found virulence genes, resistance genes were much less common in wound swabs. *parC* was the most frequently detected (11.5%), while *gyrA* was found in 3.8% of isolates. The plasmid-mediated resistance genes *qnrA*, *qnrB*, and *qnrS* were each detected in only 1.9% of isolates.

Sputum samples also had virulence genes, especially *lasB* (24.4%), *oprI* (22.2%), and *toxA* (20.0%). Resistance genes such as *gyrA* (4.4%) and *parC* (11.1%) were detected, with *qnrA* and *qnrB* each appearing in 6.7% of isolates, and *qnrS* in 4.4%.

Other sample types—including blood, tracheal aspirates, eye swabs, pus, tissue, body fluids, and cough swabs—showed a variable distribution of resistance and virulence genes. Among these, *oprI*, *lasB*, and *toxA* were detected sporadically across most sample types, with the highest frequencies observed in eye swabs (*oprI* 40.0%) and fluids (*oprI* and *lasB*, each 50.0%). The *nan1* gene was generally absent in these samples, except for tracheal aspirates (8.3%) and blood (6.2%). Qnr genes were largely absent or rare in these samples: they were entirely undetected in blood, eye swabs, fluids, pus, and tissue samples, and appeared only occasionally in tracheal aspirates (*qnrB* and *qnrS*, each 8.3%) and RF samples (*qnrB* and *qnrS*, each 20.0%).

## Discussion

This study offers a detailed examination of the prevalence and distribution of virulence and ciprofloxacin resistance genes in clinical *P*. *aeruginosa* isolates and their implications for ciprofloxacin resistance.

The *oprI* gene had been identified in 88.4% of our isolates, which is in line with previous studies reporting prevalence rates between 55% and 91.1% [14,15]. This range of results might be due to differences in location, the types of clinical samples, or the diagnostic methods used to determine the gene. Still, the fact that *oprI* is consistently found at high rates shows that it is a common and conserved virulence factor in *P. aeruginosa*.

In our investigation, 80.2% of isolates had the *lasB* gene, which is a bit lower than the rates seen in earlier studies (93.3% to 100%) [16,17]. These differences could be due to variations in where the isolates came from, differences in the patient population, or environmental factors that affect how the gene is expressed and maintained. Even so, the high detection rate in our study highlights how important elastase B is in the pathogenicity of *P. aeruginosa*.

For the *toxA* gene, the 57% prevalence observed is in line with some studies that reported higher rates (92% and 69.4%) [17,18] but contrasts with reports that reported lower rates (15% and 32.4%) [14,15]. This variability highlights the complexity of *P. aeruginosa* pathogenicity and underscores the need for standardized approaches in gene prevalence reporting.

6.98% of the isolates demonstrated the *nan1* gene, so it’s significantly lower than the prevalence reported by Hassan Abdulaali Al-Saeedi et al. [19]. This difference may be due to regional, patient, or methodological factors. The presence of multiple virulence factors in a single isolate supports the notion that *P. aeruginosa* can harbor several virulence determinants, increasing its pathogenic potential [17,18]. However, it is important to interpret these findings with caution, particularly those involving rare genes such as *nan1*. Because there were only a few isolates in these subgroups, our analyses may not have had sufficient statistical power, and other factors may still be influencing the results. Future studies with larger and more diverse samples will be necessary to confirm these findings and gain a deeper understanding of the clinical significance of these rare genetic markers

The observed ciprofloxacin resistance rate of 34.9% aligns with several studies (44.19%, 41.37%, and 59.4%) [20–22] but contrasts with the lower resistance rate (20.6%) reported in a previous Sudanese study and the significantly higher rate (97%) reported in another study [23,24]. This variability reflects the dynamic nature of antimicrobial resistance and suggests that local factors and differences in study design may play a role. In our study, gender was not significantly associated with ciprofloxacin resistance in *P.aeruginosa* (p = 0.798), with an odds ratio of 0.889 (95% CI: 0.361–2.191). These findings are consistent with previous research indicating that gender does not independently predict resistance in *P. aeruginosa* infections [25]. Antimicrobial resistance in *P. aeruginosa* is more commonly influenced by antibiotic exposure history, genetic resistance determinants, and hospital-acquired factors rather than patient demographics [26]. However, further large-scale studies are warranted to explore potential gender-specific trends in resistance patterns.

We noticed a strong connection between the hospital unit and ciprofloxacin resistance in *P.aeruginosa* isolates (p = 0.015). Resistance was most common in intensive care units, with the main ICU showing 43.3% and the male ICU 30.0% of strains resistant to ciprofloxacin. On the other hand, we didn’t find any resistant strains in the emergency room, outpatient clinics, pediatric, or operating room units. It’s not surprising to see resistance clustering in critical care settings, since these areas often involve more invasive treatments, frequent use of broad-spectrum antibiotics, and longer hospital stays all of which can encourage resistance to develop. Our findings are in line with local studies showing that *P. aeruginosa* from hospital-acquired infections in Sudan tends to be more resistant than strains picked up in the community. For example, Omer et al. [23] found that isolates from diabetic wound infections in two Khartoum hospitals were often resistant to ciprofloxacin and other antibiotics, highlighting just how common resistant strains are in clinical settings.

Finding resistant strains in different ICU subunits—such as ICU-ERP, ICU-RT, ICU-CNT, and PICU-RT—shows just how important it is to have strong antibiotic stewardship and infection control in these high-risk hospital areas.

Overall, these findings highlight the need for targeted monitoring of antibiotic resistance in intensive care units. This can help guide treatment choices and help prevent the spread of multidrug-resistant bacteria.

Ciprofloxacin resistance was significantly associated with the genes *gyrA*, *qnrA*, *qnrB*, and *qnrS* (p < 0.001), but not *parC*. We detected the *gyrA* and *parC* genes together in 7 (23.3%) *P. aeruginosa* ciprofloxacin-resistant isolates, which is consistent with findings from other investigations [27–29].

We found the *gyrA* and *parC* genes in isolates that were still sensitive to ciprofloxacin. However, just having these genes doesn’t mean the bacteria are resistant. Usually, resistance occurs when there are specific mutations in QRDRs of these genes. Finding these genes in susceptible isolates suggests that these important mutations haven’t happened, or that the genes haven’t changed in a way that would cause resistance. Since we didn’t perform sequencing or expression analysis, the phenotypic relevance of these findings remains inconclusive. [2]. Alternatively, resistance may be linked to the concurrent existence of *gyrA* and *parC* mutations. [21]. These findings highlight the critical role of these genes in ciprofloxacin resistance.

In our study, *qnrS* had higher rates in the qnr genes than did *qnrA* and *qnrB*, while both had the same lowest rates. In contrast, Ataei B et al. and Abdelrahim SS et al. reported that *qnrA* was more prevalent than *qnrB* and *qnrS* in all samples and that most samples contained at least one qnr gene [30,31]. Our findings are consistent with the study in which *qnrS* was also detected, whereas *qnrA* and *qnrB* were absent, in contrast to our findings [32]. Additionally, all the resistance genes identified in our study were also reported in earlier reports, although prevalence varied [22,33]. Notably, *qnrB* was more prevalent than *qnrA* and *qnrS* in those studies. We also reported that the three PMQR genes under investigation (*gnrA*, *qnrB*, and *qnrS*) coexist in 11 (36.7%) of the resistant *P. aeruginosa* isolates.

Our multivariable logistic regression analysis showed a strong and statistically significant link between the presence of the *gyrA* gene and ciprofloxacin resistance (OR = 33.5, 95% CI: 5.97–188.2, FDR-adjusted p < 0.001), highlighting the key role that chromosomal changes play in the development of resistance. This finding matches what other PCR-based study have reported, with high rates of *gyrA* found in ciprofloxacin-resistant *P. aeruginosa* from clinical samples in Iran [34].

On the other hand, the *qnrA, qnrB,* and *qnrS* genes were found only in resistant isolates and not at all in sensitive ones. Because of this perfect split, we couldn’t include these genes in our multivariable models a challenge that’s also been noted in other studies that look at gene presence or absence [22,35].

Overall, our findings suggest that ciprofloxacin resistance in these isolates is mainly linked to chromosomal changes especially the presence of the *gyrA* gene, which showed a strong connection to resistance. While we also found plasmid-mediated resistance genes (*qnrA, qnrB, qnrS*) only in resistant isolates, their consistent presence made it hard to analyze them further. Therefore, while our findings strongly support the role of chromosomal resistance, they also point to a possible contribution from plasmid-based genes that warrants further investigation.

While virulence genes such as *toxA*, *lasB*, *nan1*, and *oprI* were frequently detected among our isolates, they did not show a statistically significant association with ciprofloxacin resistance after adjusting for multiple comparisons. This suggests that these genes may contribute more to pathogenicity than to antimicrobial resistance. Similar findings have been reported by Edward et al. [36], who found no clear link between virulence gene carriage and ciprofloxacin resistance in *P. aeruginosa* isolates from burn patients.

The types of resistance and virulence genes we found in *P. aeruginosa* depended on the source of the clinical samples, with urine specimens exhibiting the highest combined prevalence of both virulence and resistance genes. In our study, *oprI* (23.5%), *lasB* (20.0%), and *toxA* (13.0%) were the most frequently detected virulence genes in urinary *P.aeruginosa* isolates far more common than nan1 (3.5%). This aligns with previous work demonstrating that *lasB* and *toxA* genes are universally present in urinary *P.aeruginosa* isolates, reinforcing the idea of site-specific virulence enrichment in urine-derived strains [37].

We also found that urine samples had relatively high rates of the resistance genes *parC* (11.3%), *gyrA* (10.4%), and *qnrA* (7.0%) indicating that urinary isolates can act as reservoirs for both plasmid-mediated quinolone resistance (PMQR) and chromosomal QRDR resistance. This is consistent with previous observations: Tehran-based research confirmed frequent co-occurrence of *gyrA* and *parC* mutations in urinary *P. aeruginosa* isolates [38], and a multicenter analysis found that *qnrA, qnrS,* and *qnrB* were prevalent among fluoroquinolone-resistant strains, particularly from urinary sources [39].

In contrast, wound swabs showed a high prevalence of virulence genes such as *lasB* (30.8%), *oprI* (26.9%), and *toxA* (21.2%), findings consistent with previous reports linking these determinants to proteolytic tissue damage, immune evasion, and persistence of *P. aeruginosa* in skin and soft tissue infections [40]. Resistance genes were much less common—*parC* (11.5%), *gyrA* (3.8%), and *qnr* genes (1.9% each for *qnrA*, *qnrB*, and *qnrS*), which aligns with earlier findings showing that plasmid-mediated quinolone resistance genes are uncommon in *P. aeruginosa* [41], This pattern may reflect localized antibiotic pressure in skin and soft tissue infections. Previous studies on wound isolates especially from burn wounds highlight have identified the *gyrA* gene as the predominant ciprofloxacin resistance mechanism in such settings [42].

Sputum samples also showed notable levels of virulence genes, with *lasB* (24.4%), *oprI* (22.2%), and *toxA* (20.0%) being the most common. This matches previous reports that highlight *toxA* as a frequent virulence factor and note the presence of *lasB* in clinical *P. aeruginosa* isolates [43]. Resistance genes appeared less common overall, although *parC* (11.1%) and *qnrA/qnrB* (6.7% each) were detected more often than *gyrA* (4.4%). This predominance of *parC* over *gyrA* contrasts with findings from a regional survey of clinical P. aeruginosa isolates including a substantial number derived from pus and sputum specimens where the *gyrA* were the most frequently observed mechanism of fluoroquinolone resistance. In that survey, alterations in *parC* were less common, and plasmid-mediated qnr genes were notably absent [44].

Samples from other sources—such as blood, tracheal aspirates, pus, tissue, body fluids, and cough swabs—displayed a variety of gene patterns. Notably, *oprI* was found in 40.0% of eye swabs and 50.0% of fluid samples. The *nan1* gene was mostly absent, except in tracheal aspirates (8.3%) and blood (6.2%). qnr genes were rarely detected outside of urine and sputum, only appearing in tracheal aspirates (*qnrB* and *qnrS*, 8.3% each) and RF samples (20.0% each), and entirely undetected in blood, eye swabs, fluids, pus, and tissue.

Interestingly, no single sample type had all the resistance or all the virulence genes at once, highlighting how much gene patterns can vary depending on the infection site. This underlines the importance of specimen-specific surveillance and intervention strategies.

These findings have important real-world implications, especially for hospital infection control and how doctors choose antibiotics to start treatment. Notably, the fact that ciprofloxacin-resistant *P. aeruginosa* strains were mostly found in intensive care units highlights the urgent need for stronger antibiotic stewardship in these high-risk areas. Intensive care units often involve more invasive procedures, longer patient stays, and frequent use of broad-spectrum antibiotics—factors that all make it easier for resistant bacteria to take hold and spread. This study set out to explore ciprofloxacin resistance as well as the presence of resistance and virulence genes, to gain a clearer picture of the molecular factors that influence clinical outcomes.

We also noticed that certain resistance and virulence genes like *gyrA, qnrS,* and *toxA* were more common in specific types of samples, such as tracheal aspirates and wound swabs. This points to the possibility of site-specific genetic adaptation, which could help make surveillance and monitoring efforts more targeted and effective.

Understanding these patterns can help healthcare teams spot high-risk infections sooner and choose better initial treatments, which in turn can lead to better patient outcomes and help slow the spread of multidrug-resistant bacteria.

A major limitation of this study is the lack of sequencing of the *gyrA* and *parC* genes, which prevents confirmation of resistance-conferring mutations. Additionally, we didn’t perform minimum inhibitory concentration (MIC) testing, which would have given us a more precise way to classify resistance. Our sample size was relatively small just 86 isolates which may affect how well our results can be applied to a broader population. In addition, we didn’t analyze gene expression, so we don’t know how active these resistance genes really are. Another important limitation is that some genes, like nan1, were only found in a small number of isolates. Because of these small group sizes, our statistical analysis power is reduced, and there’s a chance that some associations could be misleading. So, these findings should be taken with a grain of salt. While they do give us useful clues especially about how resistance and virulence traits can exist together—they are still exploratory at this stage. We recognize these limitations and plan to address them in future research.

## Conclusion

In summary, our study shows that the patterns of virulence and resistance genes in *P. aeruginosa* isolates are quite complicated. The differences seen across various studies make it clear that factors like location and research methods can really influence the results, so it’s important to keep these in mind when interpreting the findings. Continued monitoring and more advanced research are key to better understanding how resistance develops and how we can best manage *P. aeruginosa* infections. Based on what we found especially the concentration of ciprofloxacin-resistant strains in intensive care units and the links between certain genes and specific types of samples, we suggest putting targeted antibiotic stewardship programs and gene-based surveillance in place for high-risk hospital units. These findings are crucial for enhancing infection control and antibiotic use, enabling healthcare teams to respond more effectively to the challenges posed by this adaptable pathogen.

## Supporting information

S1 Data Raw dataset of gene presence/absence and clinical metadata used for statistical analysis. Provided as an Excel spreadsheet prior to statistical processing.
(XLSX)

## Abbreviations:

*P. aeruginosa*: *Pseudomonas aeruginosa*
QRDR: Quinolone Resistance Determining Region
PMQR: Plasmid-mediated quinolone resistance
DNA: Deoxyribonucleic Acid
PCR: polymerase chain reaction
